# Transcatheter aortic valve replacement with the VenusA-Pro and VenusA-Plus systems: preliminary experience in China

**DOI:** 10.3389/fcvm.2023.1169590

**Published:** 2023-08-24

**Authors:** Jie Li, Yinghao Sun, Songyuan Luo, Shengneng Zheng, Jiaohua Chen, Ming Fu, Zhenfei Fang, Yan Wang, Guang Li, Ruixin Fan, Jianfang Luo

**Affiliations:** ^1^ Guangdong Provincial People’s Hospital, Guangdong Academy of Medical Sciences, Guangdong Cardiovascular Institute, Guangzhou, China; ^2^ Department of Cardiology, The Second Xiangya Hospital of Central South University, Changsha, China; ^3^ Department of Cardiology, Xiamen Cardiovascular Hospital of Xiamen University, Xiamen, China

**Keywords:** aortic stenosis (AS), bicuspid aortic valve (BAV), transcatheter aortic valve replacement (TAVR), VenusA-Pro, VenusA-Plus

## Abstract

**Background:**

The outcomes of transcatheter aortic valve replacement (TAVR) employing the second-generation retrievable VenusA-Pro and VenusA-Plus delivery systems with the self-expanding VenusA-Valve have not been described yet. This study aims to report the outcomes of these two second-generation delivery systems.

**Methods:**

From January 2022 to April 2023, we prospectively enrolled patients with severe aortic stenosis undergoing TAVR with VenusA-Pro from three centers across China in this first-in-man study and retrospectively identified those undergoing TAVR with VenusA-Plus. All outcomes were reported according to the Valve Academic Research Consortium 3 definition. The primary outcome was 30-day all-cause mortality.

**Results:**

A total of 156 patients were included, of which 46 underwent TAVR with VenusA-Pro and 110 underwent TAVR with VenusA-Plus. The Society of Thoracic Surgeons median score was 2.1%, bicuspid anatomy prevalence rate was 55.1%, and the mean aortic root calcification volume was 693 mm^3^. The technical success rate was 91.7%, comparable between the VenusA-Pro and VenusA-Plus groups (87.0% vs. 93.6%, *P *= 0.169). The 30-day all-cause mortality was 2.6%, similar between the VenusA-Pro and VenusA-Plus groups (2.2% vs. 2.7%, *P *= 0.842). No myocardial infarction occurred. The incidences of stroke (0.6%), major bleeding (3.8%), major vascular complications (5.1%), acute kidney injury (9.0%), permanent pacemaker implantation (5.1%), new-onset atrial fibrillation (5.8%), and moderate-to-severe paravalvular aortic regurgitation (6.0%) were favorable and comparable between the two groups. The clinical outcomes were similar between the patients with bicuspid and tricuspid aortic valve, except that the incidence of permanent pacemaker implantation was lower in patients with bicuspid anatomy (1.2% vs. 10.6%, *P *= 0.010).

**Conclusions:**

The 30-day outcomes of TAVR with VenusA-Pro and VenusA-Plus were favorable and comparable.

## 1. Introduction

Transcatheter aortic valve replacement (TAVR) has emerged as an alternative to surgical aortic valve replacement in elderly patients with symptomatic severe aortic stenosis (AS) (1). The VenusA-Valve (Venus Medtech Inc., Hangzhou, China), a self-expanding valve with first-generation non-retrievable delivery system, has been widely used in TAVR (2, 3). In China, challenging TAVR cases characterized by bicuspid anatomy and severe aortic valve calcification were more prevalent than in Western countries (4–6). The VenusA-Valve has seen a relatively higher rate of valve malposition and paravalvular aortic regurgitation in such a challenging TAVR population (7), and new-generation retrievable devices may help reduce peri-procedural complications (8). The VenusA-Valve second-generation retrievable and repositionable delivery systems are the VenusA-Pro and VenusA-Plus (Venus Medtech Inc., Hangzhou, China). The first-in-man use of the VenusA-Plus system has been reported previously in a single patient, but not in a larger population (9). Here, we aimed to report the outcomes of both VenusA-Pro and VenusA-Plus second-generation delivery systems. In addition, this was the first-in-man study of the latest VenusA-Pro system.

## 2. Methods

### 2.1. Study design

From January 2022 to April 2023, we prospectively enrolled patients from three centers across China in this first-in-man study of the VenusA-Pro system. In the same time frame, we also retrospectively identified the control group comprising TAVR patients using the VenusA-Plus system in Guangdong Provincial People's Hospital. All TAVR procedures were performed in patients with symptomatic severe aortic stenosis diagnosed according to the guideline (1). The baseline characteristics, procedural characteristics, clinical outcomes, and hemodynamic outcomes within 30 days were collected. The protocols were approved by site-specific institutional review boards. All patients in the prospective cohort had provided written informed consent, but those in the retrospective cohort did not.

### 2.2. Devices

**Figure 1:**
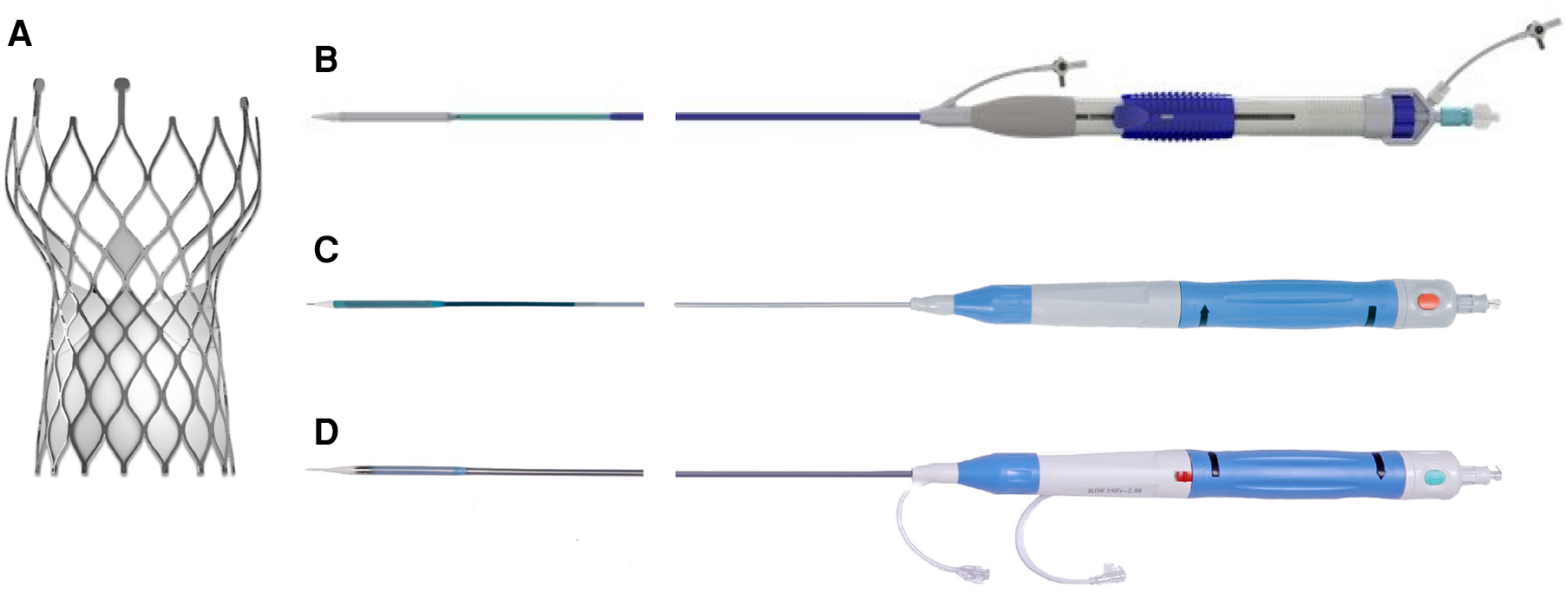
VenusA-Valve prosthesis and iteration of its delivery systems. (**A**) VenusA-Valve prosthesis, (**B**) first-generation non-retrievable delivery system, (**C**) second-generation retrievable VenusA-Plus delivery system, (**D**) second-generation retrievable VenusA-Pro delivery system. The red button in the middle of the handle is the safety lock.

**Figure 2:**
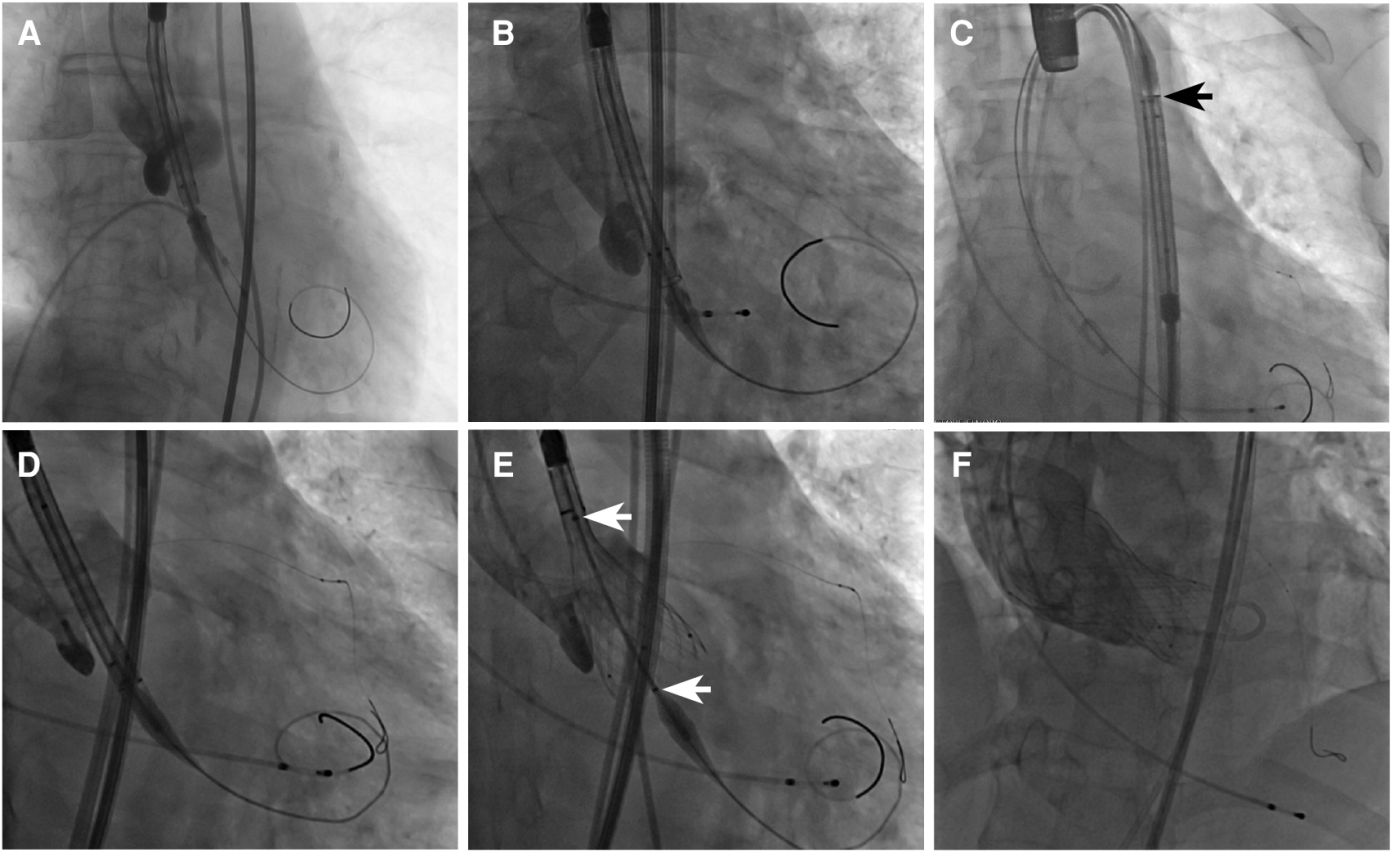
Fluoroscopic view of different delivery systems and their common VenusA-Valve prosthesis. (**A**) First-generation non-retrievable delivery system, (**B**) VenusA-Plus, and (**C**) VenusA-Pro. The black arrow shows the extra marker for orientation, which should point to the greater curve of the aortic arch. (**D**) VenusA-Pro locating the prosthesis, with left coronary artery under protection. (**E**) VenusA-Pro releasing the prosthesis after three times of retrieval and adjustment in a challenging case with a horizontal aorta with an aortic root angle of 73°. The white arrows show two limiting markers for reference during release or retrieval. (**F**) Final position of the VenusA-Valve prosthesis released by the VenusA-Pro delivery system, aortography showing trivial paravalvular aortic regurgitation.

The VenusA-Valve features supra-annular design similar to the Medtronic CoreValve (Medtronic Inc., Minneapolis, MN, USA) but with stronger radial force at the inflow end, which may be advantageous in bicuspid anatomy and severe calcification (2). Both VenusA-Pro and VenusA-Plus are second-generation retrievable and repositionable delivery systems that use the same prothesis VenusA-Valve as in the first-generation non-retrievable delivery system. The prosthesis is available in four different sizes (23, 26, 29, and 32 mm). The VenusA-Pro has the same profile as the VenusA-Plus, with an outer diameter ranging from 18 to 19 Fr. Compared to the VenusA-Plus, the VenusA-Pro offers more major advantages. First, the VenusA-Pro features a safety lock to prevent accidental valve deployment. Second, there is an additional marker for delivery system orientation for a better commissural alignment and coronary protection, which should point to the greater curve of the aortic arch. Third, the front end of the sheath has better flexibility when confronted with the horizontal aorta. Lastly, two limiting markers were used as reference during release or retrieval (Figure 1, 2).

### 2.3. Outcome definition

All outcomes were reported according to the Valve Academic Research Consortium 3 definition (10). The primary outcome was 30-day all-cause mortality. Secondary outcomes included technical success, stroke, myocardial infarction, major bleeding (type 2–4), major vascular complications, acute kidney injury (stage 2–4), permanent pacemaker implantation, new-onset atrial fibrillation, and moderate-to-severe paravalvular aortic regurgitation at a 30-day follow-up. Post-procedural hemodynamic outcomes were measured before discharge.

### 2.4. Statistical analyses

Continuous variables were presented as mean with standard deviation and median with interquartile range and were compared using the Student’s *t*-test and the Mann–Whitney *U*-test, respectively. Categorical variables were presented as percentages and compared using the chi-squared test and Fisher’s exact test. All tests were two-tailed and *P *< 0.05 was considered significant. All statistical analyses were performed using SPSS version 25.0 (SPSS Inc., Chicago, IL, USA).

## 3. Results

### 3.1. Baseline characteristics

A total of 156 patients were included, of which 46 underwent TAVR with VenusA-Pro and 110 underwent TAVR with VenusA-Plus. The mean age was 71.6 years, and 64.7% were male (Table 1). The Society of Thoracic Surgeons (STS) median score was 2.1%, which was slightly higher in the VenusA-Pro group (2.6% vs. 2.0%, *P *= 0.026). The percentage of patients with New York Heart Association (NYHA) class III or IV was lower (34.8% vs. 59.1%, *P *= 0.006), and the incidence of peripheral artery disease (defined according to the criteria in the STS score) was higher (15.2% vs. 2.7%, *P *= 0.004) in the VenusA-Pro group, whereas the incidences of other comorbidities were comparable between the two groups. The pre-procedural mean aortic valve area (0.61 vs. 0.74 cm^2^, *P *= 0.006) and annular perimeter (75.4 vs. 78.5 mm, *P *= 0.031) were smaller in the VenusA-Pro group, while other echocardiography and computed tomography characteristics were similar between the two groups. It was worth mentioning that over half of the patients (55.1%) had bicuspid anatomy, with a mean aortic root calcification volume of 693 mm^3^.

**Table 1:** Baseline characteristics.

	Total	VenusA-Pro	VenusA-Plus	*P*-value
	(*n* = 156)	(*n* = 46)	(*n* = 110)	
Clinical variables				
Age, years	71.6 ± 6.8	70.6 ± 7.9	72.0 ± 6.3	0.245
Male sex	101 (64.7)	27 (58.7)	74 (67.3)	0.307
Body mass index, kg/m^2^	23.3 ± 3.8	23.3 ± 4.2	23.4 ± 3.6	0.936
NYHA class III or IV	81 (51.9)	16 (34.8)	65 (59.1)	**0**.**006**
STS score, %	2.1 (1.5–3.7)	2.6 (1.7–4.3)	2.0 (1.4–3.4)	**0**.**026**
Hypertension	76 (48.7)	22 (47.8)	54 (49.1)	0.885
Diabetes	30 (19.2)	10 (21.7)	20 (18.2)	0.607
Coronary artery disease	41 (26.3)	13 (28.3)	28 (25.5)	0.717
Previous myocardial infarction	11 (7.1)	5 (10.9)	6 (5.5)	0.228
Previous percutaneous coronary intervention	17 (10.9)	4 (8.7)	13 (11.8)	0.568
Peripheral artery disease	10 (6.4)	7 (15.2)	3 (2.7)	**0**.**004**
Previous stroke	9 (5.8)	1 (2.2)	8 (7.3)	0.213
Atrial fibrillation	14 (9.0)	2 (4.3)	12 (10.9)	0.191
Permanent pacemaker	1 (0.6)	0 (0)	1 (0.9)	0.516
Chronic obstructive pulmonary disease	4 (2.6)	0 (0)	4 (3.6)	0.190
Chronic kidney disease (eGFR < 60 ml/min/1.73 m^2^)	47 (30.1)	9 (19.6)	38 (34.5)	0.063
Echocardiographic variables				
Aortic valve area, cm^2^	0.67 ± 0.20	0.61 ± 0.19	0.74 ± 0.19	**0**.**006**
Mean transaortic gradient, mmHg	58.2 ± 17.9	59.1 ± 18.1	57.9 ± 17.8	0.702
Peak aortic velocity, m/s	4.85 ± 0.68	4.87 ± 0.67	4.84 ± 0.69	0.776
Left ventricular ejection fraction, %	55.2 ± 13.4	53.4 ± 14.1	56.0 ± 13.1	0.273
Moderate-to-severe aortic regurgitation	66 (42.3)	18 (39.1)	48 (43.6)	0.603
MDCT variables				
Bicuspid aortic valve	86 (55.1)	26 (57.8)	60 (56.1)	0.847
Aortic root calcification volume, mm^3^	693 ± 571	745 ± 688	672 ± 517	0.471
Annular perimeter, mm	77.6 ± 8.2	75.4 ± 8.3	78.5 ± 8.0	**0**.**031**
Aortic root angle, °	48.7 ± 10.5	49.4 ± 9.9	48.3 ± 10.8	0.559

eGFR, estimated glomerular filtration rate; MDCT, multidetector computed tomography.

Data are presented as mean ± standard deviation, median (interquartile range), or *n* (%).

Bold values signify *P*<0.05.

### 3.2. Procedural characteristics

The majority of patients underwent the procedure using general anesthesia (93.6%) and transfemoral access (94.2%) (Table 2). The rates of combined percutaneous coronary intervention, pre-dilation, and post-dilation in all patients were 13.5%, 98.7%, and 48.7%, respectively, which were comparable between the two groups. The prostheses sizes were generally smaller in the VenusA-Pro group (*P *= 0.019). The mean perimeter oversizing was 5.7%, which was comparable between the two groups. The second valve implantation rate was 1.9% in all patients without a significant difference between the two groups. Two patients (1.3%) had tamponade. Two patients (1.3%) were transferred to open surgery, one with the VenusA-Pro was due to annular rupture, while the other one with the VenusA-Plus was due to valve embolization to the ascending aorta. No coronary obstruction occurred. The technical success rate was 91.7%, comparable between the VenusA-Pro and VenusA-Plus groups (87.0% vs. 93.6%, *P *= 0.169).

**Table 2:** Procedural characteristics.

	Total	VenusA-Pro	VenusA-Plus	*P*-value
	(*n* = 156)	(*n* = 46)	(*n* = 110)	
General anesthesia	146 (93.6)	45 (97.8)	101 (91.8)	0.162
Transfemoral access	147 (94.2)	45 (97.8)	102 (92.7)	0.213
Combined percutaneous coronary intervention	21 (13.5)	7 (15.2)	14 (12.7)	0.678
Pre-dilation	154 (98.7)	46 (100.0)	108 (98.2)	0.357
Post-dilation	76 (48.7)	20 (43.5)	56 (50.9)	0.397
Prostheses size, mm^a^				**0** **.** **019**
23	41 (26.5)	17 (37.8)	24 (21.8)	
26	78 (50.3)	23 (51.1)	55 (50.0)	
29	32 (20.6)	3 (6.7)	29 (26.4)	
32	4 (2.6)	2 (4.4)	2 (1.8)	
Perimeter oversizing, %	5.7 ± 7.8	6.2 ± 8.6	5.4 ± 7.5	0.561
Second valve implantation	3 (1.9)	2 (4.3%)	1 (0.9)	0.154
Tamponade	2 (1.3)	1 (2.2)	1 (0.9)	0.522
Coronary obstruction	0 (0)	0 (0)	0 (0)	NA
Conversion to open surgery	2 (1.3)	1 (2.2)	1 (0.9)	0.522
Technical success^b^	143 (91.7)	40 (87.0)	103 (93.6)	0.169

Data are presented as mean ± standard deviation or *n* (%).

^a^ Prosthetic valve was not implanted in one patient in the VenusA-Pro group.

^b^ Technical success was adjudicated at exit from procedure room.

Bold values signify *P*<0.05.

### 3.3. Thirty-day clinical outcomes

The 30-day all-cause mortality was 2.6%, similar between the VenusA-Pro and VenusA-Plus groups (2.2% vs. 2.7%, *P *= 0.842) (Table 3). Three patients died due to cardiogenic shock, and one died due to septic shock and subsequent multiple organ dysfunction syndrome. No myocardial infarction occurred. The incidence of stroke (0.6%), major bleeding (3.8%), major vascular complications (5.1%), acute kidney injury (9.0%), permanent pacemaker implantation (5.1%), and new-onset atrial fibrillation (5.8%) were favorable and comparable between the VenusA-Pro and VenusA-Plus groups.

**Table 3:** Thirty-day clinical outcomes.

	Total	VenusA-Pro	VenusA-Plus	*P*-value
	(*n* = 156)	(*n* = 46)	(*n* = 110)	
Death	4 (2.6)	1 (2.2)	3 (2.7)	0.842
Stroke	1 (0.6)	0 (0)	1 (0.9)	0.516
Myocardial infarction	0 (0)	0 (0)	0 (0)	NA
Major bleeding (type 2–4)	6 (3.8)	3 (6.5)	3 (2.7)	0.261
Major vascular complications	8 (5.1)	4 (8.7)	4 (3.6)	0.191
Acute kidney injury (stage 2–4)	14 (9.0)	2 (4.1)	12 (9.9)	0.191
Permanent pacemaker implantation	8 (5.1)	3 (6.5)	5 (4.5)	0.610
New-onset atrial fibrillation	9 (5.8)	4 (2.7)	5 (6.3)	0.311

NA, not available.

Data were presented as *n* (%).

### 3.4. Post-procedural hemodynamic outcomes

The rates of moderate-to-severe paravalvular aortic regurgitation was 6.0% in all patients, without significant difference between the VenusA-Pro and VenusA-Plus groups (2.2% vs. 7.6%, *P *= 0.202). The post-procedural mean aortic valve area (1.72 cm^2^), mean transaortic gradient (12.0 mmHg), peak aortic velocity (2.14 m/s), and left ventricular ejection fraction (56.6%) were also similar between the two groups (Table 4).

**Table 4:** Post-procedural hemodynamic outcomes.

	Total	VenusA-Pro	VenusA-Plus	*P*-value
	(*n* = 150)	(*n* = 45)	(*n* = 105)	
Aortic valve area, cm^2^	1.72 ± 0.54	1.70 ± 0.50	1.73 ± 0.56	0.874
Mean transaortic gradient, mmHg	12.0 ± 5.2	12.4 ± 6.4	11.8 ± 4.6	0.585
Peak aortic velocity, m/s	2.14 ± 0.59	2.15 ± 0.65	2.14 ± 0.56	0.889
Left ventricular ejection fraction, %	56.6 ± 12.3	56.0 ± 13.1	56.9 ± 12.0	0.706
Moderate-to-severe aortic regurgitation	9 (6.0)	1 (2.2)	8 (7.6)	0.202

Data are presented as mean ± standard deviation or *n* (%).

### 3.5. Comparison between bicuspid and tricuspid aortic valve

In this study, based on the pre-procedural computed tomography analysis, 86 (55.1%) patients had bicuspid aortic valve (BAV), 66 had tricuspid aortic valve, three had bioprosthetic aortic valve failure, and one lacked pre-procedural computed tomography due to emergency (Table 5). The patients with bicuspid aortic valve were younger (70.5 vs. 73.3 years, *P *= 0.005) and had lower incidence of coronary artery disease (18.6% vs. 36.4%, *P *= 0.014). The pre-procedural mean transaortic gradient (62.6 vs. 53.4 mmHg, *P *= 0.002) and peak aortic velocity (5.03 vs. 4.65 m/s, *P *= 0.001) were higher, whereas the incidence of moderate-to-severe aortic regurgitation was lower (33.7% vs. 53.0%, *P *= 0.017) in patients with bicuspid aortic valve. The aortic root calcification volume was higher in patients with BAV (836 vs. 507 mm^3^, *P *< 0.001) than those with tricuspid aortic valve. During TAVR procedure, post-dilation was performed more frequently in patients with bicuspid aortic valve (55.8% vs. 39.4%, *P *= 0.045). The prosthesis sizes were generally smaller in patients with BAV than those with tricuspid aortic valve (*P *= 0.044). The mean perimeter oversizing was smaller in patients with BAV than those with tricuspid aortic valve (2.8% vs. 8.6%, *P* <0.001). The technical success rates were similar between the two groups (91.9% vs. 90.9%, *P *= 0.835). The incidence of permanent pacemaker implantation was lower in patients with BAV than those with tricuspid aortic valve (1.2% vs. 10.6%, *P *= 0.010). There was no significant difference in 30-day all-cause mortality (1.2% vs. 4.5%, *P *= 0.197) and other clinical outcomes between patients with bicuspid and tricuspid aortic valve. The post-procedural mean transaortic gradient (12.7 vs. 10.5 mmHg, *P *= 0.031) and peak aortic velocity (2.21 vs. 2.02 m/s, *P *= 0.049) was higher in patients with bicuspid aortic valve. The incidence of moderate-to-severe paravalvular aortic regurgitation were similar between patients with bicuspid and tricuspid anatomy (6.0% vs. 6.5%, *P *= 0.901).

**Table 5:** Comparison between patients with bicuspid and tricuspid aortic valve.

	Bicuspid	Tricuspid	*P*-value
	(*n* = 86)	(*n* = 66)	
Baseline characteristics clinical variables			
Age, years	70.5 ± 6.0	73.3 ± 6.4	**0**.**005**
Male sex	56 (65.1)	42 (63.6)	0.850
Body mass index, kg/m^2^	23.4 ± 3.7	23.6 ± 3.9	0.727
NYHA class III or IV	47 (54.7)	32 (48.5)	0.451
STS score, %	1.8 (1.4–3.3)	2.4 (1.5–4.1)	0.079
Hypertension	39 (45.3)	36 (54.5)	0.261
Diabetes	18 (20.9)	12 (18.2)	0.673
Coronary artery disease	16 (18.6)	24 (36.4)	**0**.**014**
Previous myocardial infarction	5 (5.8)	6 (9.1)	0.440
Previous percutaneous coronary intervention	7 (8.1)	9 (13.6)	0.274
Peripheral artery disease	4 (4.7)	6 (9.1)	0.274
Previous stroke	5 (5.8)	4 (6.1)	0.949
Atrial fibrillation	7 (8.1)	6 (9.1)	0.835
Permanent pacemaker	0 (0)	1 (1.5)	0.252
Chronic obstructive pulmonary disease	2 (2.3)	2 (3.0)	0.788
Chronic kidney disease (eGFR <60 ml/min 1.73 m^2^)	21 (24.4)	24 (36.4)	0.110
Echocardiographic variables			
Aortic valve area, cm^2^	0.63 ± 0.21	0.73 ± 0.18	0.056
Mean transaortic gradient, mmHg	62.6 ± 18.2	53.4 ± 16.2	**0**.**002**
Peak aortic velocity, m/s	5.03 ± 0.64	4.65 ± 0.68	**0**.**001**
Left ventricular ejection fraction, %	54.7 ± 13.6	56.5 ± 12.3	0.385
Moderate-to-severe aortic regurgitation	29 (33.7)	35 (53.0)	**0**.**017**
MDCT variables			
Aortic root calcification volume, mm^3^	836 ± 649	507 ± 382	**<0**.**001**
Annulus perimeter, mm	78.9 ± 8.4	76.6 ± 7.1	0.082
Aortic root angle, °	49.7 ± 10.0	47.6 ± 11.0	0.221
Procedural characteristics			
General anesthesia	83 (96.5)	59 (89.4)	0.079
Transfemoral access	81 (94.2)	63 (95.5)	0.728
Combined percutaneous coronary intervention	8 (9.3)	12 (18.2)	0.108
Pre-dilation	85 (98.8)	65 (98.5)	0.850
Post-dilation	48 (55.8)	26 (39.4)	**0**.**045**
Prostheses size^a^			**0**.**044**
23 mm	29 (34.1)	10 (15.2)	
26 mm	38 (44.7)	38 (57.6)	
29 mm	15 (17.6)	17 (25.8)	
32 mm	3 (3.5)	1 (1.5)	
Perimeter oversizing, %	2.8 ± 7.0	8.6 ± 6.9	**<0**.**001**
Second valve implantation	1 (1.2)	2 (3.0)	0.412
Coronary obstruction	0 (0)	0 (0)	NA
Conversion to open surgery	0 (0)	2 (3.0)	0.104
Technical success	79 (91.9)	60 (90.9)	0.835
Thirty-day clinical outcomes			
Death	1 (1.2)	3 (4.5)	0.197
Stroke	1 (1.2)	0 (0)	0.379
Myocardial infarction	0 (0)	0 (0)	NA
Major bleeding (type 2–4)	1 (1.2)	5 (7.6)	0.086
Major vascular complications	6 (7.0)	2 (3.0)	0.280
Acute kidney injury (stage 2–4)	5 (5.8)	8 (12.1)	0.168
Permanent pacemaker implantation	1 (1.2)	7 (10.6)	**0**.**010**
New-onset atrial fibrillation	5 (5.8)	4 (6.1)	0.949
Post-procedural hemodynamic outcomes^b^			
Aortic valve area, cm^2^	1.63 ± 0.53	1.82 ± 0.55	0.174
Mean transaortic gradient, mmHg	12.7 ± 5.5	10.5 ± 4.3	**0**.**031**
Peak aortic velocity, m/s	2.21 ± 0.63	2.02 ± 0.51	**0**.**049**
Left ventricular ejection fraction, %	57.5 ± 11.1	56.0 ± 13.6	0.440
Moderate-to-severe aortic regurgitation	5 (6.0)	4 (6.5)	0.901

Data were presented as mean ± standard deviation, median (interquartile range) or *n* (%).

^a^ Prosthetic valve was not implanted in one patient in the bicuspid aortic valve and VenusA-Pro groups, as mentioned in Table 2.

^b^ Only 146 patients (84 with bicuspid and 62 with tricuspid aortic valve) who had echocardiography after TAVR were analyzed.

Bold values signify *P*<0.05.

## 4. Discussion

**Figure 3:**
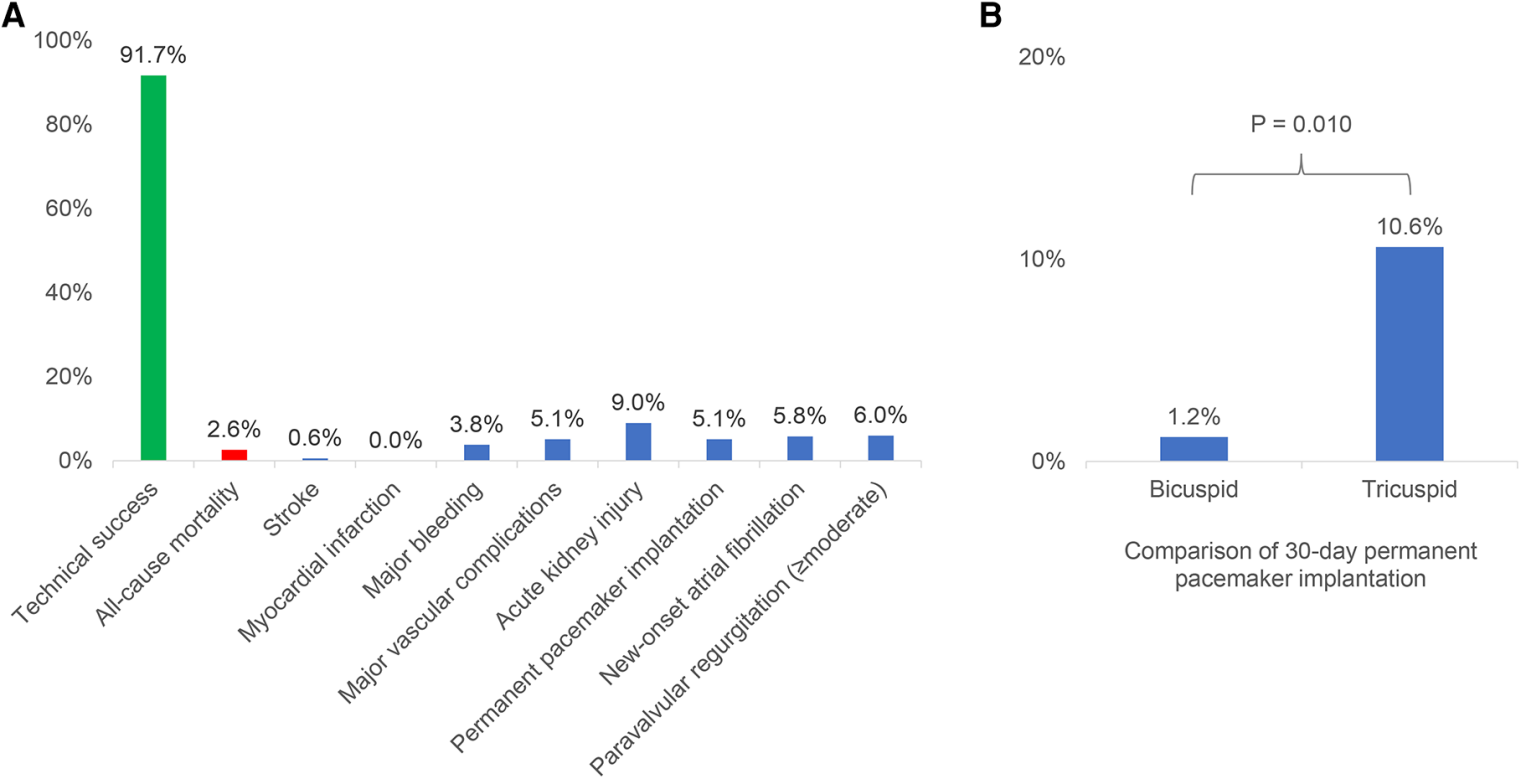
The 30-day outcomes of transcatheter aortic valve replacement with the VenusA-Pro and VenusA-Plus. (**A**) Incidences of 30-day outcomes of the VenusA-Pro and VenusA-Plus groups. The outcomes were favorable and comparable between the two groups. (**B**) Comparison of 30-day permanent pacemaker implantation between the patients with bicuspid and tricuspid aortic valve, the incidence was lower in patients with bicuspid anatomy.

This was a first-in-man multicenter study of the VenusA-Pro system and the largest report of the VenusA-Plus system so far. The main findings included the following: (1) the 30-day outcomes of TAVR with VenusA-Pro and VenusA-Plus were favorable and comparable, (2) the 30-day clinical outcomes of TAVR with both second-generation delivery systems were similar between bicuspid and tricuspid aortic valve, except that the incidence of permanent pacemaker implantation was lower in those with bicuspid anatomy (Figure 3).

In this study of two second-generation retrievable delivery systems using the self-expanding VenusA-Valve prosthesis, the overall 30-day mortality was 2.6%, with no significant difference between the VenusA-Pro and VenusA-Plus groups. The 30-day mortality of the most widely used retrievable self-expanding Evolut R (Medtronic, Minneapolis, MN, USA) was around 3.4% (8), similar to our results. The 30-day mortality of our study was also acceptable compared to that (1.9%) of the latest-iteration self-expanding Evolut PRO/PRO+ (Medtronic, Minneapolis, MN, USA) in the OPERA-TAVI registry (11).

With regard to procedural characteristics, the prosthesis sizes were generally smaller in the VenusA-Pro group as compared to the VenusA-Plus group, which could be explained by a smaller baseline aortic valve area and annular perimeter. The overall technical success rate in our study (91.7%) was comparable to that (93.1%) of the latest-iteration self-expanding Evolut PRO/PRO+ (11).

With respect to peri-procedural complications, the risk of stroke or myocardial infarction in this study was low, similar to that of the latest-iteration self-expanding valves (11). The rates of bleeding and vascular complications in this study were reasonable compared to previous reports (8, 12). The overall risk of acute kidney injury was 9.0% in this study, numerically lower than the 30-day incidence (17%) reported in a BRAVO-3 trial substudy (13), and similar to that (6.0%) reported in a previous review of Evolut R (8). The incidence of permanent pacemaker implantation was 5.1% in our study, generally lower than those reported in the previous studies (11, 14, 15), which may be explained by a downsizing strategy and a higher release position of the valve and subsequently less compression on the conduction system in patients with bicuspid anatomy with severe calcification. The overall incidence of new-onset atrial fibrillation in this study (5.8%) was numerically lower than that (9.9%) reported in a previous review (16).

As for hemodynamic performance, the post-procedural mean transaortic gradient in this study (12.0 mmHg) was slightly higher than that of the Evolut PRO/PRO+ (7.0 mmHg), and this might be explained by a lower perimeter oversizing rate (5.7%) as compared to that (18.9%) in a previous study (11). As was shown in the baseline characteristics, over half of the patients had bicuspid anatomy, and the mean aortic root calcification volume was near 700 mm^3^, indicating a population of challenging anatomy. Considering the strong radial force of the VenusA-Valve (though released by second-generation retrievable systems) in this study, the downsizing strategy (17) was frequently used to avoid severe valve migration toward the ventricle and subsequent unacceptable paravalvular aortic regurgitation. The rate of moderate-to-severe paravalvular aortic regurgitation was 6.0% in this study, slightly higher than that (3.2%) of the Evolut PRO/PRO+ (11), which might be explained by the absence of a VenusA-Valve external wrap, downsizing strategy, and high calcium burden (possibly bulky calcification) at the aortic root in our study. Actually, three generations of the Medtronic CoreValve System have seen lower incidence of moderate-to-severe paravalvular aortic regurgitation in a matched population, with 8.3% in CoreValve, 5.4% in Evolut R, and 3.4% in Evolut PRO (*P *= 0.032) (18). As paravalvular aortic regurgitation has been proved to be a risk factor on short-term and long-term mortality (19), further efforts should be made to minimize its incidence and severity.

The proportion of bicuspid aortic valve in this study was over 50%, similar to that (48.5%) reported in a previous study from China (6), and much higher than in other countries (4). The mean aortic root calcification volume was higher in patients with BAV than those with tricuspid aortic valve (836 vs. 507 mm^3^), both of which were higher than that (382 mm^3^) reported in Western patients with bicuspid aortic valve stenosis undergoing TAVR (20). The higher calcium burden could account for generally smaller prostheses, a higher post-dilation rate (55.8% vs. 39.4%), and a higher post-procedural mean transaortic gradient (12.7 vs. 10.5 mmHg) and peak aortic velocity (2.21 vs. 2.02 m/s) in patients with bicuspid anatomy. Previous studies have shown comparable survival rates after TAVR in patients with bicuspid and tricuspid anatomy (21, 22), similar to our findings. Nevertheless, excessive calcification has been related to poorer outcomes in patients with bicuspid anatomy (20). It was worth mentioning that the risk of permanent pacemaker implantation was significantly lower in patients with bicuspid anatomy (1.2% vs. 10.6%), which might be explained by a downsizing strategy as reflected by a lower perimeter oversizing rate (2.8% vs. 8.6%) and also by a higher release strategy, both of which were frequently used in severely calcified bicuspid anatomy.

There were several limitations in this study. First, there can be selection bias in such an observational study. Second, the statistic power maybe insufficient considering the small sample size, unmeasured confounding factors, and unbalanced baseline characteristics. Lastly, long-term outcomes were not available. Future studies with larger sample size and longer follow-up are warranted to further testify our findings.

## 5. Conclusions

In this study of the VenusA-Pro and VenusA-Plus delivery systems using the self-expanding VenusA-Valve, we found that the 30-day outcomes of TAVR with VenusA-Pro and VenusA-Plus were favorable and comparable (2). The clinical outcomes of both second-generation delivery systems were similar between patients with bicuspid and tricuspid aortic valve, except that the incidence of permanent pacemaker implantation was lower in patients with bicuspid anatomy.

## Data Availability

The raw data supporting the conclusions of this article will be made available by the authors, without undue reservation.
